# Mental health during the Covid-19 pandemic: reports from health professionals in Rio de Janeiro

**DOI:** 10.11606/s1518-8787.2026060006425

**Published:** 2026-04-17

**Authors:** Débora Castanheira, Carla Pereira, Thiago Silva Torres, Daniele Novaes, Gessica Ferreira, Thiago Jacobino, Thaís Regis Aranha Rossi, Valdilea Gonçalves Veloso

**Affiliations:** I Fundação Oswaldo Cruz. Instituto Nacional de Infectologia Evandro Chagas. Rio de Janeiro, RJ, Brasil; II Universidade Federal da Bahia. Instituto de Saúde Coletiva. Salvador, BA, Brasil

**Keywords:** COVID-19, Health Professionals, Mental Health, Primary Health Care, Areas of Poverty

## Abstract

**OBJECTIVE:**

To explore the perception of health professionals who worked in primary health care between 2020 and 2022, in the Manguinhos neighborhood of Rio de Janeiro, regarding the risk and protective factors for their mental health during the management of the Covid-19 pandemic.

**METHODS:**

This is a qualitative study integrated into a broader investigation on testing strategies, quarantine, digital health, and telemonitoring in areas of high socioeconomic vulnerability in Brazil. Three focus groups were conducted with community health agents and ten semistructured interviews with higher education professionals. The data were collected between November 2022 and March 2023. The analysis was conducted based on the psychodynamics of work, and the procedures followed the content analysis technique.

**RESULTS:**

The study identified several factors that contributed to high levels of stress and anxiety among health professionals who worked in family health units during the Covid-19 pandemic. The main reported factor was the constant fear of death. Additionally, work overload, lack of adequate personal protective equipment, feelings of decision-making incapacity, and absence of mental health support exacerbated the impact on the professionals’ health. Among community health agents, the perception of exclusion concerning the health professionals category was particularly noted.

**CONCLUSION:**

The Covid-19 pandemic revealed the urgent need to prioritize the mental health of health professionals from the onset of any health crisis. Preparing for future emergencies requires proactive actions, such as equitable distribution of workload, adequate provision of personal protective equipment, and free and accessible access to specialized mental health treatment for health professionals.

## INTRODUCTION

The Covid-19 pandemic revealed profound challenges for health systems, especially in countries like Brazil^1^, exposing fragility in human, financial, and infrastructure resources. Given that similar crises, intensified by climate change, may become more common, it is essential to learn from the pandemic to strengthen the resilience of health systems and ensure more effective responses in the future.

The pandemic period deeply impacted health professionals (HP), causing effects on their mental health^2,3^ and on work processes^4,5^. The combination of high mortality rates, rapid transmission of the disease, and fragilities in health systems significantly affected the well-being of health professionals, effects that continue to unfold^6^.

Studies have reported the impact of the pandemic on the mental health of health professionals as a consequence of work overload, shortage of personal protective equipment (PPE), lack of testing, and fear of being infected and/or transmitting the infection^3,7,8^. Although most studies focus on urgent or emergency services^2,3,9^, two studies showed that health professionals in primary health care (PHC) suffered from anxiety, depression, and emotional trauma as a result of the pandemic^10,11^.

The situation is more serious when the PHC is located in areas of high socioeconomic vulnerability^12^, a common occurrence in Rio de Janeiro. During the pandemic, HP in PHC were confronted with unprecedented circumstances. Recognizing that work is central to the formation of identity and the mental health of the worker^13^, this study aims to explore the perception of HP who worked during the Covid-19 pandemic in a community in Rio de Janeiro regarding risk and protective factors for their mental health.

## METHODS

### Study Design

This study is part of the project "Expansion of testing, quarantine, digital health, and telemonitoring strategies to address the Covid-19 pandemic in Brazil," which involved 19 PHC units in areas of high socioeconomic vulnerability in Salvador (BA) and Rio de Janeiro (RJ)^14^. This study addressed the data related to Rio de Janeiro.

This is a qualitative study with a socio-anthropological approach, developed through focus groups (FG) with community health agents (CHA) and semistructured interviews (SI) with other higher education professionals (HEP) between November 2022 and March 2023. The experiences of HP from two Family Health Units (FHU) during the Covid-19 pandemic (2020–2022) in the Manguinhos neighborhood were considered.

Eight SI were conducted with HEP in the FHU and two SI with professionals from the Municipal Health Department of Rio de Janeiro (SMS-RJ) responsible for territorial management. The SIs took place at the FHUs or remotely, depending on the convenience of the participants. Three in-person FG were conducted with CHA in the two FHUs. It was decided to conduct FG with CHA and SI with HEP due to the impossibility of gathering enough HEP for a FG without compromising care. To preserve anonymity, each participant received a code composed of the type of data collection (SI or FG), a code referring to the FHU (A or B), a numerical identifier for which FG (1, 2, or 3), and a unique participant number. The results were presented separately for HEP and CHA when there were divergences in the perception of the relationships between work and mental health.

The SIs and FG addressed the perception of the impact of Covid-19 on the personal and professional sphere of HP; the management of health services in the municipality of Rio de Janeiro; and the impacts on the lives of residents of Manguinhos (Chart 1).

**Chart 1:** Themes addressed in the questions of the semi-structured interviews and focus groups.

Theme	Approached content
1. Significant effects of the Covid-19 pandemic	Confinement/Social distancing; Impact on work in FHUs; Fear of CHAs and HEPs working during the Covid-19 pandemic.
2. Knowledge about Covid-19	Understanding of virus transmission; Access to personal protective equipment (PPE); Access to information; Vaccine efficacy .
3. Impacts of Covid-19 on health services and the response to the pandemic in the city of Rio de Janeiro	Difficulties faced; Physical distancing or lack thereof from users in FHUs; PPE for professionals on the front lines of the pandemic; Suggestions for improving epidemiological surveillance; Infrastructure of FHU; Number of people in the teams.
4. Testing	Training; FHU organization; Access to testing by professionals and users; Resistence and difficulty to conduct them; Differentiation of test types.
5. Services related to Covid-19 in FHUs	Surveillance – monitoring of positive cases and information about Covid-19; Distribution of PPE to users and healthcare professionals; Screening and reception of suspected cases; Referral and counter-referral; Vaccination; Promotion of FHU services to the community; Protocols.
6. Consequences of Covid-19 in the communities of Manguinhos	Implementation or non-implementation of social distancing; Implementation or non-implementation of isolation for positive cases (difficulties in enforcing isolation); Impact of receiving emergency assistance; Recommended and adopted protective measures by the population in the communities; Sources of information for decision-making to protect against the virus (including fake news).

HP: health professional; FHU: Family Health Unit; CHA: community health agents.

### Selection of Participants

The selection criteria were: (1) HP (physicians, nurses, dentists, and CHA) that worked during 2020–2021 in one of the FHUs or linked to the Municipal Health Department of Rio de Janeiro (SMS-RJ); (2) involvement in the response to Covid-19 in planning, testing, and/or vaccination. A maximum variation sampling strategy was used, considering the professional category of the HP and their workplace. When no new information emerged and the identified themes were adequately grounded, the invitation to participate was concluded.

### Theoretical Reference

The dynamics between work and mental health are analyzed from the perspective of Work Psychodynamics (WPD). According to WPD, work is central to the formation of identity and the mental health of the worker, and it can be a source of suffering or pleasure^13^. By analyzing the produced data, WPD was used to identify how the HP perceive their work, what their needs are, and which factors they perceive as risks and protections for their mental health.

The content analysis described by Bardin^16^, was used to clarify the processes of meaning-making and the themes that emerged from the participants’ statements. For Bardin^16^, there are three fundamental phases in content analysis: (1) pre-analysis (organization and initial contact with the material); (2) exploration of the material (coding, classification, and categorization); (3) treatment of the results (inference and interpretation).

### Definition of Categories and Data Analyses

The SIs and FG were transcribed, and a floating reading and in-depth re-reading were conducted to construct the categories/analysis axes. In the exploration of the material, thematic axes for analysis were established to organize the units of record according to all aspects considered in the SI and FG scripts, and the data were systematized into four main analysis categories and subcategories, using the software MAXQDA (v. 2022). Chart 2 shows the results of this classification regarding the themes presented in the article.

**Chart 2:** Indicators of the effects and impacts of the Covid-19 pandemic on the personal and professional lives of the interviewees^a^.

Categories of analysis	Subcategories	Content of subcategories
1. Effects of the Covid-19 pandemic on the personal lives of HP.	Fear of infection	Fear of the new virus generating panic and anxiety; Working on the front lines, raising concerns about Covid-19 infection; Fear of infecting family members living in the same household (taking precautions with clothes upon returning home and distancing from others); Concern about possible deaths of coworkers; Fear of one's own death; Questions about Covid-19 transmission; Learning to cope with fears and insecurities (support from protocols); Tension in managing fear and the deaths of service users; Decision-making regarding which user would receive the first care and oxygen; Lack of protective and care supplies (PPE, oxygen, among others); Difficulty for users in wearing masks; Challenges in referring and counter-referring users to hospitals; Difficulty in obtaining ambulances for user transfers; Discrimination and prejudice from neighbors who did not want contact with HP and community health agents from the FHU; Pressure for decision-making regarding users, leading professionals to rapidly develop new skills; Psychological pressure causing binge eating, but regulated by physical exercise; No mental rest for professionals and CHAs at home, as they remained worried about users (deaths and hospitalizations) and coworkers; Pressure from FHU managers to maintain work schedules, especially for CHAs; Aggressiveness from users who arrived for care.
2. Impacts of the Covid-19 pandemic on the work process of FHUs	Work overload	Working on the front lines, raising concerns about Covid-19 infection; Screening in external spaces to avoid crowding, resulting in physical exhaustion and generating tension (hours spent standing, in the sun, and experiencing fainting); Role changes for HEPs and CHAs; Removal of CHAs and HEPs due to Covid-19; Large number of users requiring transfer to hospitals at the peak of the pandemic; Working in hospitals that treated severe Covid-19 cases, impacting the mental health of HEPs; Lack of infrastructure in FHUs to address Covid-19; CHAs and HEPs with comorbidities removed from work; Testing for HEPs to confirm diagnoses; Vaccination as a form of protection and safety at work; Differentiation between CHAs and HEPs (provision of PPE, bonuses, lack of vacations, etc.); Constant changes in technical standards (which needed to be checked daily); Leaving and/or requesting leave due to psychological issues; Not recovering mental health three years after the Covid-19 pandemic (development of depression and anxiety); Focusing on work as a means of escaping reality and the loneliness at home.
2. Impacts of the Covid-19 pandemic on the work process of FHUs	Feeling of helplessness	Attempts to calm users of health services, especially those with more severe cases; Provision of adequate PPE after the increase in the number of deaths, primarily for CHAs; Reorganization of services, which did not prevent user deaths due to difficulties in transferring them to hospitals; Transformation of HEP's work into "emergency responders" or "intensivists"; Death of users and lack of space/places to store bodies; Need for HEPs to stay in health services at night when they could not find beds in hospitals for users; Lack of testing for CHAs and HEPs at the beginning of the pandemic, as well as the withdrawal from health services due to Covid-19 infection; CHAs using the knowledge acquired in health services to care for family members with Covid-19, especially those with comorbidities; Vaccination as an indicator of preventing the worsening of Covid-19, emphasized by HEPs to CHAs; Increased attention to hygiene upon arriving home, especially after returning from work or when infected with Covid-19; Pressure felt by HEPs who also worked in Covid-19 hospitals; Fear of the new virus, which could cause their own death and that of users in health services; Tension between the Family Health Unit (CFVV) and the nearby Emergency Care Unit (UPA), which could not attend to severe cases referred by the health service due to overcrowding at the peak of the pandemic; Difficulty in counter-referring users sent to field hospitals, as HEPs attempted to obtain information to relay to the families of hospitalized individuals; Fear of the death of users and their families; Change in the routine of primary care (CHAs) with differentiated work due to Covid-19; CHAs witnessing the deaths of acquaintances, friends, and relatives in health services; Change in the work of CHAs, becoming restricted to health services.
	Lack of mental health actions	Attempts by SMS-RJ to provide psychological support for CHAs and HEPs; Late provision of support by psychology professionals; Non-institutionalized actions for mental health support for CHAs and HEPs; Differentiation in mental health support for HEPs versus lack of support for CHAs; Provision of mental health assistance for HEPs via phone, online psychologists, etc., but which did not reach CHAs from CSEGSF; Lack of mental health assistance for CHAs;

a Not all the points listed were developed in the article. CHA: community health agent; CFVV: Family Clinic Victor Valla; CSEGSF: School Health Center Germano Sinval Faria; PPE: personal protective equipment; HEP: higher education professional; SMS-RJ: Municipal Health Secretariat of Rio de Janeiro; UPA: Emergency Care Unit; FHU: Family Health Unit.

We address the categories related to mental health in the results: (1) Effects of the Covid-19 pandemic on the personal lives of professionals; (2) Impacts of the Covid-19 pandemic on the work process of the FHU (Figure).

**Figure:**
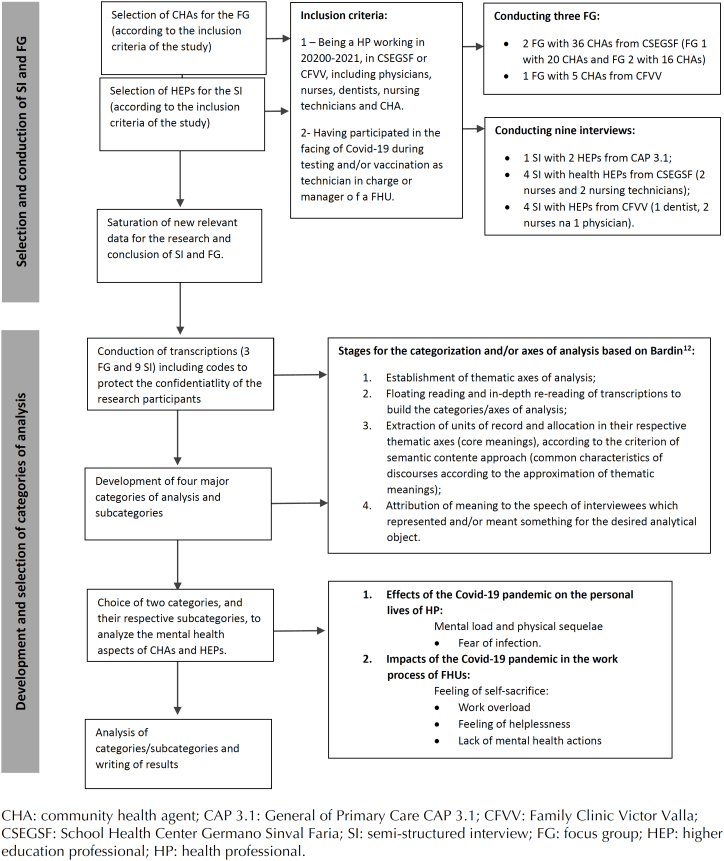
Flowchart of selection/conduction of semistructured interviews/focus groups and development/selection of categories of analysis.

### Ethical Considerations

The study was approved by the Research Ethics Committee of the Instituto Nacional de Infectologia – INI/Fiocruz (CAE n^o^53844121.4.3001.5240).

## RESULTS

Most participants were female (90%), aged 35–59 years (73%), pardos (mixed-race) (43%), and had completed high school (51%). The percentage of women was higher for CHA (95%) than among HEP (70%), as well as the proportion of black/pardo individuals (73% versus 40%), as indicated in Table.

**Table:** Sociodemographic profile of higher education professionals and community health agents who participated in the research. Rio de Janeiro, RJ, 2022–2023.

		FG		SI		Total	
		n	%	n	%	n	%
		41	100	10	100	51	100
Sex							
	Female	39	95.1	7	70.0	46	90.2
	Male	2	4.9	3	30.0	5	9.8
Occupation							
	CHA	41	100	0	0	41	80.4
	Nurse	0	0	7	70.0	7	13.7
	Physician	0	0	2	20.0	2	3.9
	Dentist	0	0	1	10.0	1	2.0
Race/ethnicity							
	White	11	26.8	6	60.0	17	33.3
	Black	10	24.4	2	20.0	12	23.5
	Pardo (mixed-race)	20	48.8	2	20.0	22	43.1
Age group (years)							
	25–34	8	19.5	2	20.0	10	19.6
	35–44	14	34.1	5	50.0	19	37.3
	45–59	15	36.6	3	30.0	18	35.3
	≥ 60	4	9.8	0	0	4	7.8
Schooling							
	Complete High School	26	63.4	0	0	26	51.0
	Incomplete Higher Education	6	14.6	0	0	6	11.8
	Complete Higher Education	7	17.1	4	40.0	11	21.5
	Specialization/postgraduation	2	4.9	6	60.0	8	15.7

FG: focus groups; SI: semi-structured interview; CHA: community health agents.

The professionals reported intense and multifaceted psychological suffering, described as emotional exhaustion, a sense of helplessness, and physical and mental overload. Psychological suffering was portrayed as a combination of chronic stress, insomnia, and irritability, with some professionals reporting difficulties in balancing work demands and personal care. All of this led, according to the HP, to an increase in cases of anxiety and depression.

### Effects of the Covid-19 Pandemic on the Personal Lives of Healthcare Professionals

#### "[…] I was sure I was going to die": fear of infection.

The reports from the HP highlight a constant fear of death. Initially, this feeling manifested as a concern for their own health, primarily due to a lack of information about the disease and the scarcity of PPE for work:

"[…] there was a very strong fear of getting infected and losing your family […]. I think that was the most striking part. Being there caring for others while at the same time being afraid of losing your own life […]" (ESA03).

The fear of the CHA arose primarily from changes in their roles to increase the capacity of healthcare services in combating Covid-19, which made them responsible for coordinating the flow of patients and directing them for triage in the units, frequently dealing with symptomatic patients who were not wearing masks. Additionally, they received PPE that they considered incompatible with their role and inferior to that provided to HEPs, such as surgical masks instead of N95 masks, and gowns with a weight below that recommended by the Agência Nacional de Vigilância Sanitária (Anvisa — National Health Surveillance Agency) for PPE^17^.

Although PPE and safety protocols reduced insecurities after 2021, the fear of death persisted. Concern about infecting relatives led professionals to adopt safety measures upon arriving home, especially those with children or elderly family members. These measures included sanitizing their clothes and body before entering and, in extreme cases, sending children and elderly relatives to stay with family members.

The fear of patients dying also affected the mental health of HP, resulting in panic and anxiety crises. The CHAs living in Manguinhos witnessed the daily deaths from Covid-19 of neighbors and relatives. This made work in the FHU "unsustainable":

"[…] in my case, I really left because I was already feeling very unstable psychologically, I was completely sure I was going to die. I said, "I'm going to die." It was either that or have a psychotic break" (GFA0209).

"People are dying […]. And the panic, you know? And the anxiety crisis already shaking you, and you not knowing what to do […]. It was very complicated. And we were on the front lines, not knowing if we […] would be the next, the next victim, you know?" (GFB0101).

This fact was exacerbated by the behavior of the users themselves, who arrived fearful of death at the healthcare services. According to the reports, the professionals had to try to "stay strong" to help, even while being "afraid of losing their own lives," and ensure that the patients would be okay:

"We would arrive and a patient who would say: "please, don't intubate me because I don't want to die," and by the end of the day, he would die" (ESB03).

The feeling of fear of death was diminished by the start of vaccination for HP:

"So, I felt very relieved when I was able to get vaccinated, but at the same time very saddened for the two people I lost who didn't have the opportunity to take it. And it was very close; one was about a week before getting vaccinated, and the other was about two weeks after, something like that" (GFA0205).

Despite this, many participants reported that the delay in the purchase and availability of the vaccine also caused anxiety:

"The vaccination process took a long time. So, in order to protect the professionals and have them at the front lines to work, it took time. It was a lot of anxiety, very tense, waiting" (ESB02).

At the time of the research, three years after the onset of the pandemic, there were still reports from HEP who had not recovered from the psychological pressure of the more intense years of Covid-19:

"The mental health of workers, really, even today we still see that some people have not returned to their normalcy, you know? […]. We are seeing many people still experiencing panic, developing post-pandemic depression… […] We are seeing people who still haven't recovered, who have really become ill" (ESA01).

### Impacts of the Covid-19 Pandemic on the Work Process of FHU

#### "Primary care ended up experiencing a huge overload": work overload

Another factor impacting mental health was the increase in workload during the pandemic due to longer hours, the removal of more vulnerable professionals, and the frequent dual workload (PHC and emergencies), leading to burnout in many professionals.

This led to an increase in absenteeism/leave, overburdening those who remained. In the perception of HP, the removal of CHAs and HEPs due to comorbidities and Covid-19 infection resulted in reduced teams, increasing the workload of the remaining staff.

In addition, CHAs reported, at the time of data collection, that they had gone years without taking a vacation due to the change in social organization, the non-profit private entity that operates public health services on behalf of the government, contracted by SMS-RJ. The continuous work affected the mental health of this group, increasing the pressure they felt to maintain their work schedules. Those who were away also experienced changes in their sleep routine and a lack of time for rest, as even at home, there was concern for the patients who were hospitalized and mourning for those who had died. These thoughts prevented the CHAs and HEPs from disconnecting from work.

The uncertainty of the technical guidelines, which required study outside of regular working hours for constant adaptation by HP, increased the workload. Sudden changes in the criteria for conducting tests, how to receive patients, and the use of PPE were common. For CHAs, physical exhaustion resulting from long hours outdoors, in the sun, and standing while triaging patients at the FHU included reports of fainting.

#### "We began to see a different reality that primary care did not see": feeling of helplessness

The pandemic caused severely ill patients to seek care at the FHU, leading HP to face a reality for which they were not prepared. The work exceeded the functions that HEPs typically performed in the services, which was exhausting and required knowledge beyond that related to primary care. It was common to attend to severe cases, make decisions to prevent death, provide follow-up to hospitals, and deal with the lack of beds in their daily work. Months of work under these conditions negatively affected the mental health of the HEPs:

"From a psychological standpoint, there was a lot of stress at work. There were moments when we had to make decisions […] that should not have been part of primary care. […] I was no longer able to sustain being in that situation and truly wanting to give up" (ESB04).

The lack of hospital beds made admissions difficult and led many more severe patients to be treated at the FHU themselves. According to reports, this type of situation was common during 2020 and 2021, worsening during peak hospitalization periods. Healthcare professionals reported feeling as if they were losing control of the situation, increasing their anxiety and work-related stress.

Another important point was the lack of and/or difficulty in accessing ambulances for transferring patients who needed intensive treatment, with one HEP using their own car to take a patient to the hospital. In one of the FHUs there were cases of the healthcare team spending the night with patients due to the lack of Covid-19 beds in the municipality. This also exhausted the HEPs, who did everything possible to prevent patient deaths, especially during the first wave.

In addition, the professionals were confronted with the death of patients. For the CHAs, the deaths of users in primary care were more shocking because they did not work in hospitals and therefore did not see people dying repeatedly or being intubated, which had implications for the mental health of this group. Furthermore, the people who died were often friends or relatives, another factor that had a negative impact.

The perception of HEPs was that their role changed with the Covid-19 pandemic, primarily due to the need for rapid care of patients requiring first aid to prevent death. Those working in Covid-19 hospitals handled more of these cases, as they had more experience, and were compared to "emergency responders" or "intensivists" (outside the scope of primary care). Therefore, these professionals also passed on the knowledge they acquired in hospitals to those who did not have such skills.

For the CHAs, the pandemic meant a change in their work process, with a reduction in home visits, restricted only to high-risk groups and needing to be brief and limited to the in the surroundings of the household (front, sides, and back of the house). The CHAs were assigned to bureaucratic tasks at the FHU, under the argument that moving around the territory made them potential spreaders of the virus. This was considered a deviation from their role by the professionals, who felt a certain nostalgia for the type of previous work and resented the "bureaucratization" of their functions.

#### "We also didn't have support. We didn't have anyone to turn to": lack of mental health actions

The joint reports from HEPs and CHAs indicate that SMS-RJ did not prioritize the mental health of HP during the Covid-19 pandemic. According to the HEPs, there were reports of attempts by SMS-RJ to offer some support through telemedicine and follow-ups, which, in many cases, were not conducted by psychologists. Support from these professionals began to be offered, according to the HEPs, late in the pandemic and only toward the end:

"I realized that this follow-up ended up being somewhat late. When they finally looked at the professionals, it was closer to the end of the pandemic, when things were already different" (ESB01).

The FHUs created informal spaces and activities to support the mental health of healthcare professionals, such as conversations with colleagues. However, these initiatives were not institutionalized. The few available mental health care activities were not widely accessed by HEPs due to work shifts that made it difficult to seek specialized attention.

The CHAs reported feeling excluded from mental health care, as they only received information about activities afterward. For them, this exacerbated the deeper issue of not being considered full healthcare professionals.

"They said there were phones, online psychologists, and various actions available, and that we were only called for presentation. But we were not mentioned, nor were these resources utilized for us" (GFA0113).

## DISCUSSION

The results of our study indicated that work overload, fear of infection, lack of resources, and loss of control over the work environment contributed to mental suffering among HP during the Covid-19 pandemic. There is also a clear need to provide psychological support, training PPE, and promote changes in the organization of work from the outset of a health crisis.

We also found that the effects of the Covid-19 pandemic on the mental health of HP persist even after its end, as noted in international studies^18-20^. The psychological suffering of HP can impact not only their well-being but also their job performance, the quality of care, and the safety of service users^18^. The impact of mental health effects is an important factor that must be considered in the planning of health policies for HP in Rio de Janeiro.

Studies in Brazil^7,8,21^ and in other countries^11,22,23^ also indicated that the fear of death was a crucial factor, exacerbated by the perceived limitations of PPE, the lack of Covid-19 testing for HP^7,8,24^, and the delay in vaccinations. Thus, the scarcity or poor quality of PPE, particularly among CHAs, heightened the anxiety associated with the possibility of infection. The proximity to the suffering and death of patients led to psychological distress and mental illness among HP^8,24,25^, amplifying a sense of professional and civic responsibility among them^18,26^. Supporting the results of this research, the review study found that the fear of infecting a loved one was one of the main sources of distress during the Covid-19 pandemic, as highlighted by 15 articles^6^.

Work overload also negatively impacted the mental health of HP^27^, especially when they took on various roles related to pandemic control^22,27,28^, indicating that the issue is not only contact with the virus but also excessive work. Alongside the workload was the feeling of losing control of the situation, especially regarding the severity of patients, which was also found in a study conducted in Iran^2^. The large number of patients, the high hospitalization rate, the elevated mortality, and the fact that there was nothing they could do to save these patients increased the sense of loss of control^7^.

Regarding the work process, the sources of suffering differed between HEPs and CHAs. While the HEPs struggled with adapting to the severity of cases, the need to quickly acquire new knowledge, and the feeling of loss of control, the CHAs felt a greater bureaucratization of their work, with changes in their roles. An important finding was the sense of exclusion of the CHAs from the HP category. There are studies suggesting that CHA feel like the weakest link in the multidisciplinary team and are poorly recognized professionally by management and the SMS^29^, however, this topic is not well explored. Additionally, the CHA reported that pre-existing issues, such as the deterioration of labor relations, intensified during the pandemic^29^.

The results of our study on healthcare professionals in primary care highlight challenges like those faced by emergency and urgent care professionals during the pandemic. Just like in primary care, emergency professionals reported work overload, fear of infection, and insufficient resources for patient treatment, factors that intensified mental suffering^30^. However, there are notable differences in the experiences reported by HP in different contexts. While primary care workers faced significant challenges due to the scarcity of PPE and professional undervaluation, professionals in emergency units often dealt with overload and stress due to the continuous care of critically ill patients^31,32^.

Limitations include: the fact that data collection was conducted only in the Manguinhos Complex in Rio de Janeiro; the absence of FG with HEPs, which may have restricted the diversity of perspectives collected, especially on issues related to management and clinical practices; the predominance of CHAs in the sample, a group that exhibits significant differences from HEPs in terms of race and education, giving greater weight to the experiences and challenges faced by this group; and the data collection occurring after the most critical periods of the pandemic, which may have influenced the reported perceptions, reflecting a moment of adaptation and recovery rather than the more acute situations faced during the crisis.

Our results highlighted the need to prioritize the mental health of HP from the outset of a health crisis. Preparing for future health emergencies involves proactively addressing these issues, minimizing stress factors through adequate workload distribution, provision of PPE, and access to free mental health services. The experience with Covid-19 demonstrates that preparedness for health emergencies must be ongoing, with evidence-based contingency plans, coordination, communication, support for workers, and investment in the production of supplies.

## Availability Data:

The data are available upon request from the corresponding author.
