# Supplementary material for: Mental health during the Covid-19 pandemic: reports from health professionals in Rio de Janeiro
Source: Rev Saude Publica. 2026 Apr 17;60(Suppl 1):e12s. doi: 10.11606/s1518-8787.2026060006425 (PMC13089372; doi:10.11606/s1518-8787.2026060006425)
Supplement: Translation

# Translation: Saúde mental na pandemia de Covid-19: relatos de profissionais de saúde no Rio de Janeiro

**Authors:** Débora Castanheira, Carla Pereira, Thiago Silva Torres, Daniele Novaes, Gessica Ferreira, Thiago Jacobino, Thaís Regis Aranha Rossi, Valdilea Gonçalves Veloso

**Affiliations:** I Fundação Oswaldo Cruz. Instituto Nacional de Infectologia Evandro Chagas. Rio de Janeiro, RJ, Brasil; II Universidade Federal da Bahia. Instituto de Saúde Coletiva. Salvador, BA, Brasil

**Keywords:** COVID-19, Pessoal de Saúde, Saúde Mental, Atenção Primária à Saúde, Áreas de Pobreza

## Abstract

**OBJETIVO:**

Explorar a percepção de profissionais de saúde (PS) que atuaram na atenção primária à saúde entre 2020 e 2022, no bairro de Manguinhos, Rio de Janeiro, sobre os fatores de risco e proteção para sua saúde mental durante o manejo da pandemia de Covid-19.

**MÉTODOS:**

Trata-se de um estudo qualitativo integrado a uma investigação mais ampla sobre estratégias de testagem, quarentena, saúde digital e telemonitoramento em áreas de alta vulnerabilidade socioeconômica no Brasil. Foram realizados três grupos focais com agentes comunitários de saúde e dez entrevistas semiestruturadas com profissionais de nível superior. Os dados foram coletados entre novembro de 2022 e março de 2023. A análise foi conduzida com base na psicodinâmica do trabalho, e os procedimentos seguiram a técnica de análise de conteúdo.

**RESULTADOS:**

O estudo identificou diversos fatores que contribuíram para altos níveis de estresse e ansiedade entre os profissionais de saúde que atuaram em unidades de saúde da família durante a pandemia da Covid-19. O principal fator relatado foi o medo constante da morte. Adicionalmente, sobrecarga de trabalho, falta de equipamentos de proteção individual adequados, sensação de incapacidade decisória e ausência de suporte em saúde mental agravaram o impacto sobre a saúde dos profissionais. Entre os agentes comunitários de saúde, destacou-se a percepção de exclusão em relação à categoria de profissionais de saúde.

**CONCLUSÃO:**

A pandemia de Covid-19 revelou a necessidade urgente de priorizar a saúde mental dos profissionais de saúde desde o início de qualquer crise sanitária. Preparar-se para emergências futuras exige ações proativas, como a distribuição equitativa da carga de trabalho, o fornecimento adequado de equipamento de proteção individual e o acesso gratuito e acessível a tratamento especializado em saúde mental para todos os profissionais de saúde.

## INTRODUÇÃO

A pandemia de Covid-19 revelou desafios profundos para os sistemas de saúde, especialmente em países como o Brasil^1^, expondo a fragilidade em recursos humanos, financeiros e de infraestrutura. Dado que crises semelhantes, intensificadas pelas mudanças climáticas, podem se tornar mais comuns, é essencial aprender com a pandemia para fortalecer a resiliência dos sistemas de saúde e garantir respostas mais eficazes no futuro.

O período pandêmico impactou profundamente os profissionais de saúde (PS), ocasionando efeitos em sua saúde mental^2,3^ e nos processos de trabalho^4,5^. A combinação de alta taxa de mortalidade, rápida transmissão da doença e fragilidades nos sistemas de saúde afetou significativamente o bem-estar dos PS, efeitos que continuam a desdobrar-se^6^.

Estudos relataram impacto da pandemia na saúde mental dos PS como consequência da sobrecarga de trabalho, escassez de equipamentos de proteção individual (EPIs), falta de testes e medo de serem infectados e/ou transmitirem a infecção^3,7,8^. Apesar de a maior parte dos estudos ser voltada para serviços de urgências ou emergências^2,3,9^, dois estudos mostraram que PS da atenção primária à saúde (APS) sofreram com ansiedade, depressão e trauma emocional como resultado da pandemia^10,11^.

A situação é mais grave quando as APS ficam em áreas de alta vulnerabilidade socioeconômica^12^, fato comum no Rio de Janeiro. Durante a pandemia, os PS em APS foram confrontados com circunstâncias sem precedentes. Reconhecendo que o trabalho é central para a formação da identidade e para a saúde mental do trabalhador^13^, este estudo objetiva explorar a percepção de PS que atuaram durante a pandemia de Covid-19 em uma comunidade do Rio de Janeiro sobre fatores de risco e proteção para sua saúde mental.

## MÉTODOS

### Desenho do Estudo

Este estudo faz parte do projeto "Ampliação das estratégias de testagem, quarentena, saúde digital e telemonitoramento para o enfrentamento da pandemia de Covid-19 no Brasil", que envolveu 19 unidades da APS em áreas de alta vulnerabilidade socioeconômica em Salvador (BA) e no Rio de Janeiro (RJ)^14^. Neste trabalho foram abordados os dados relativos ao Rio de Janeiro.

Trata-se de um estudo de abordagem qualitativa, com base socioantropológica, desenvolvido por meio de grupos focais (GF) com agentes comunitários de saúde (ACS) e entrevistas semiestruturadas (ES) com outros profissionais de nível superior (PNS) entre novembro de 2022 e março de 2023. Foram contempladas as experiências vividas durante a pandemia de Covid-19 (2020–2022) por PS de duas Unidades de Saúde da Família (USF) do bairro de Manguinhos.

Realizaram-se oito ES com PNS nas USF e duas ES com profissionais da Secretaria Municipal de Saúde do Rio de Janeiro (SMS-RJ) responsáveis pela gestão territorial. As ES ocorreram nas USF ou remotamente, conforme a conveniência dos participantes. Foram conduzidos três GF presenciais com ACS nas duas USF. Optou-se por realizar GF com ACS e ES com PNS devido à impossibilidade de reunir PNS o suficiente para um GF sem comprometer o atendimento. Para preservar o anonimato, cada participante recebeu um código composto pelo tipo de coleta de dados (ES ou GF), código referente à USF (A ou B), identificação numérica de qual GF (1, 2 ou 3) e um número único do participante. Os resultados foram apresentados separadamente para PNS e ACS quando houve divergências na percepção das relações entre trabalho e saúde mental.

As ES e os GF abordaram a percepção do impacto da Covid-19 na esfera pessoal e profissional dos PS; a gestão dos serviços de saúde no município do Rio de Janeiro; e os impactos sobre a vida dos moradores de Manguinhos (Quadro 1).

**Quadro 1:** Temas abordados nas perguntas das entrevistas semiestruturadas e dos grupos focais.

Tema	Conteúdo abordado
1. Efeitos marcantes da pandemia de Covid-19	Confinamento/distanciamento social; Impacto no trabalho nas USF; Medo de ACS e PNS de trabalhar durante a pandemia de Covid-19.
2. Conhecimentos sobre a Covid-19	Entendimentos sobre a transmissão do vírus; Acesso aos Equipamentos de Proteção Individual (EPI); Acesso às informações; Eficácia da vacina.
3. Impactos da Covid-19 nos serviços de saúde e no enfrentamento da pandemia na cidade do Rio de Janeiro	Dificuldades enfrentadas; Distanciamento físico ou não de usuários nas USF; Medidas de proteção (EPI) para profissionais na linha de frente da pandemia; Sugestões para melhoria da vigilância epidemiológica; Infraestrutura das USF; Número de pessoas nas equipes.
4. Testagem	Capacitações; Organização das USF; Acesso ao teste pelos profissionais e usuários; Resistência e dificuldade para a sua realização; Diferenciação dos tipos de testes.
5. Serviços relacionados à Covid-19 nas USF	Vigilância – monitoramento dos casos positivos e informações sobre a Covid-19; Distribuição de EPI para usuários e PS; Triagem e acolhimento de casos suspeitos; Referência e contrarreferência; Vacinação; Divulgação dos serviços das USF para a comunidade; Protocolos.
6. Consequências da Covid-19 nas comunidades de Manguinhos	Realização ou não do distanciamento social; Realização ou não do isolamento dos casos positivos (dificuldades em realizar o isolamento); Impacto do recebimento dos auxílios emergenciais; Medidas de proteção recomendadas e adotadas pela população nas comunidades Fontes de informação para tomada de decisão para proteção do vírus (incluindo *fake news*)

PS: Profissional de Saúde; USF: Unidade de Saúde da Família; ACS: agentes comunitários de saúde; EPI: equipamento de proteção individual.

### Seleção dos Participantes

Como critérios de seleção: (1) PS (médico, enfermeiro, dentista e ACS) durante 2020–2021 em uma das USF ou vinculado à SMS-RJ; (2) participação do enfrentamento da Covid-19 no planejamento, testagem e/ou vacinação. Utilizou-se estratégia de amostragem de variação máxima^15^, considerando a categoria profissional dos PS e o local de trabalho. Quando não surgiram novas informações e os temas identificados foram devidamente fundamentados, o convite para participação foi finalizado.

### Referencial Teórico

A dinâmica entre trabalho e saúde mental é analisada a partir da abordagem da Psicodinâmica do Trabalho (PDT). Para a PDT, o trabalho é central para a formação da identidade e para a saúde mental do trabalhador e pode ser fonte de sofrimento ou prazer^13^. Ao analisar os dados produzidos, a PDT foi utilizada para identificar como os PS percebem seu trabalho, quais são suas necessidades e quais fatores percebem como risco e proteção para sua saúde mental.

Foi utilizada a análise de conteúdo descrita por Bardin^16^, buscando explicitar os processos de significação e temáticas que emergiram das falas dos participantes. Para Bardin^16^, há três fases fundamentais na análise de conteúdo: (1) pré-análise (organização e primeiro contato com o material); (2) exploração do material (codificação, classificação e categorização); (3) tratamento dos resultados (inferência e interpretação).

### Definição das Categorias e Análise dos Dados

As ES e os GF foram transcritos e foram realizadas a leitura flutuante e a releitura aprofundada para a construção das categorias/eixos de análise. Na exploração do material, foram estabelecidos eixos temáticos de análise para organizar as unidades de registro de acordo com todos os aspectos considerados nos roteiros de ES e GF, e os dados foram sistematizados em 4 grandes categorias de análise e subcategorias, utilizando o *software* MAXQDA (v. 2022). O Quadro 2 mostra o resultado dessa classificação no que concerne aos temas apresentados no artigo.

**Quadro 2:** Indicativo dos efeitos e impactos da pandemia de Covid-19 na vida pessoal e profissional dos entrevistados^a^.

Categorias de análise	Subcategorias	Conteúdo das subcategorias
1. Efeitos da pandemia de Covid-19 na vida pessoal dos PS	Medo da infecção	Medo do novo vírus gerando pânico e ansiedade; Trabalho na linha de frente, suscitando preocupação com a infecção de Covid-19; Medo de infectar parentes que moravam na mesma casa (cuidados com a roupa quando chegavam em casa e afastamento do convívio); Preocupação com possíveis mortes de colegas de trabalho; Medo da própria morte; Dúvidas sobre a transmissão da Covid-19; Aprendizagem para lidar com medos e inseguranças (auxílio dos protocolos); Tensão em lidar com o medo e com a morte de usuários dos serviços; Escolha de qual usuário iria receber o primeiro atendimento e oxigênio Falta de insumos de proteção e atendimento (EPI, oxigênio, entre outros); Dificuldade de usuários em usar máscara; Dificuldade de referência e contrarreferência de usuários para hospitais; Dificuldade de ambulâncias para transferência de usuários; Discriminação e preconceito de vizinhos, que não queriam contato com PS e ACS das USF; Pressão para a tomada de decisão de usuários, fazendo com que PS desenvolvessem novas habilidades de forma rápida; Pressão psicológica causando compulsão alimentar, mas regulada com exercícios físicos; Não descanso mental para profissionais e ACS que estavam em casa, pois ficavam preocupados com os usuários (mortes e internações) e com os colegas de trabalho; Pressão dos gestores das USF para a manutenção das escalas de trabalho, principalmente para ACS; Agressividade de usuários que chegavam para o atendimento.
2. Impactos da pandemia de Covid-19 no processo de trabalho das USF	Sobrecarga de trabalho	Trabalho na linha de frente, suscitando preocupação com a infecção de Covid-19; Triagem em espaço externo para evitar aglomeração, havendo um desgaste físico e gerando tensão (horas em pé, no sol e com desmaio); Mudança de função de PS e ACS; Afastamento de ACS e PNS em função da Covid-19; Grande quantidade de usuários que necessitavam de transferência para hospitais no pico da pandemia; Trabalho em hospitais que atendiam casos graves de Covid-19, impactando a saúde mental de PNS; Falta de estrutura das USF para o enfrentamento da Covid-19; ACS e PNS com comorbidades afastados do trabalho; Testagem para PNS para confirmação do diagnóstico; Vacinação como forma de proteção e segurança no trabalho; Diferenciação entre ACS e PNS (recebimento de EPI, gratificação, falta de férias etc.); Mudanças constantes das normas técnicas (devendo ser checadas diariamente); Saída e/ou pedido de licença por questões psicológicas; Não recuperação da saúde mental após três anos da pandemia de Covid-19 (desenvolvimento de depressão e ansiedade); Foco no trabalho utilizado como fuga da realidade e solidão do lar.
2. Impactos da pandemia de Covid-19 no processo de trabalho das USF	Sensação de incapacidade	Tentativas de acalmar usuários dos serviços de saúde, sobretudo aqueles com casos mais graves; Liberação de EPI adequado após o aumento do número de mortes, principalmente para ACS; Reorganização dos serviços, não impedindo as mortes de usuários em função da dificuldade de transferências para hospitais; Transformação do trabalho de PNS em "emergencistas" ou "intensivistas"; Morte de usuários e falta de espaço/lugar para colocar os corpos; Necessidade de PNS ficarem nos serviços de saúde à noite quando não achavam leitos em hospitais para usuários; Falta de teste para ACS e PNS no início da pandemia, como o afastamento dos serviços de saúde pela infecção de Covid-19; ACS utilizando o conhecimento adquirido nos serviços de saúde para o cuidado de familiares com Covid-19, sobretudo aqueles com comorbidade; Vacinação como indicativo do não agravamento da Covid-19, ressaltado por PNS para ACS; Maior atenção à higiene quando chegavam em casa, principalmente quando retornavam do trabalho ou quando eram infectados com a Covid-19; Pressão sentida pelos PNS que também trabalhavam em hospitais de Covid-19; Medo do novo vírus, que poderia causar a própria morte e a de usuários nos serviços de saúde; Tensão entre a CFVV e a UPA que ficava ao lado, pois não conseguia atender os casos graves encaminhados pelo serviço de saúde em função da lotação no auge da pandemia; Dificuldade de contrarreferência de usuários encaminhados para os hospitais de campanha, sendo que os PNS tentavam conseguir informações para repassar para os familiares das pessoas internadas; Medo da morte de usuários e de seus familiares; Mudança na rotina da APS, com trabalho diferenciado em função da Covid-19; ACS vendo a morte de conhecidos, amigos e parentes nos serviços de saúde; Mudança do trabalho dos ACS, ficando restritos aos serviços de saúde.
	Falta de ações de saúde mental	Tentativas da SMS-RJ de ofertar apoio psicológico para ACS e PNS; Oferta tardia de suporte por profissionais de psicologia; Ações não institucionalizadas de apoio à saúde mental para ACS e PNS; Diferenciação do auxílio à saúde mental para PNS *versus* falta de apoio para ACS; Oferta de ajuda à saúde mental para PNS via telefone, psicólogos *online* etc., mas que não chegou até os ACS da CSEGSF; Falta de assistência à saúde mental para ACS;

a Nem todos os pontos listados foram desenvolvidos no artigo. ACS: agente comunitário de saúde; CFVV: Clínica da Família Victor Valla; CSEGSF: Clínica Saúde Escola Germano Sinval Faria; EPI: equipamento de proteção individual; PNS: profissional de nível superior; SMS-RJ: Secretaria Municipal de Saúde do Rio de Janeiro; UPA: Unidade de Pronto Atendimento; USF: Unidade de Saúde da Família.

Abordamos nos resultados as categorias relacionadas à saúde mental: (1) Efeitos da pandemia de Covid-19 na vida pessoal dos profissionais; (2) Impactos da pandemia de Covid-19 no processo de trabalho das USF (Figura).

**Figura:** Fluxograma de seleção/realização de entrevistas semiestruturadas/grupos focais e desenvolvimento/seleção das categorias de análise.

### Considerações Éticas

O estudo foi aprovado pelo Comitê de Ética em Pesquisa do Instituto Nacional de Infectologia – INI/Fiocruz (CAE n^o^53844121.4.3001.5240).

## RESULTADOS

A maioria dos participantes era do sexo feminino (90%), com idade de 35–59 anos (73%), parda (43%), e com ensino médio completo (51%). O percentual de mulheres foi maior entre ACS (95%) do que entre PNS (70%), assim como de pessoas pretas/pardas (73% *versus* 40%), conforme indicado na Tabela.

**Tabela:** Perfil sociodemográfico dos profissionais de nível superior e agentes comunitários de saúde que participaram da pesquisa. Rio de Janeiro, RJ, 2022–2023.

		GF		ES		Total	
		n	%	n	%	n	%
		41	100	10	100	51	100
Sexo							
	Feminino	39	95,1	7	70,0	46	90,2
	Masculino	2	4,9	3	30,0	5	9,8
Ocupação							
	ACS	41	100	0	0	41	80,4
	Enfermeiro(a)	0	0	7	70,0	7	13,7
	Médico(a)	0	0	2	20,0	2	3,9
	Dentista	0	0	1	10,0	1	2,0
Raça/cor							
	Branco	11	26,8	6	60,0	17	33,3
	Preto	10	24,4	2	20,0	12	23,5
	Pardo	20	48,8	2	20,0	22	43,1
Faixa etária (anos)							
	25–34	8	19,5	2	20,0	10	19,6
	35–44	14	34,1	5	50,0	19	37,3
	45–59	15	36,6	3	30,0	18	35,3
	≥60	4	9,8	0	0	4	7,8
Escolaridade							
	Ensino médio completo	26	63,4	0	0	26	51,0
	Nível superior incompleto	6	14,6	0	0	6	11,8
	Nível superior completo	7	17,1	4	40,0	11	21,5
	Especialização/pós-graduação	2	4,9	6	60,0	8	15,7

GF: grupo focal; ES: entrevista semiestruturada; ACS: agentes comunitários de saúde.

Os profissionais relataram sofrimento psicológico intenso e multifacetado, descrito como exaustão emocional, sensação de desamparo e sobrecarga física e mental. O sofrimento psicológico foi retratado como uma combinação de estresse crônico, insônia e irritabilidade, com alguns profissionais relatando dificuldades para equilibrar demandas do trabalho e cuidados pessoais. Tudo isso levou, de acordo com os PS, ao aumento de quadros de ansiedade e depressão.

### Efeitos da Pandemia de Covid-19 na Vida Pessoal dos Profissionais de Saúde

#### "[…] tinha certeza que eu ia morrer": medo da infecção.

Os relatos dos PS evidenciam medo constante da morte. Inicialmente, este sentimento se apresentava como uma preocupação com a própria saúde, principalmente em razão da falta de informação sobre a doença e a falta de EPI para o trabalho:

"[…] era um medo, né, muito grande também de você pegar e você perder sua família […]. Era isso, acho que, mais marcante, foi isso. De você tá ali cuidando e ao mesmo tempo com medo de você perder sua própria vida […]" (ESA03).

O temor dos ACS vinha, sobretudo, das mudanças de função para aumentar a capacidade dos serviços de saúde no enfrentamento da Covid-19, o que os colocou como responsáveis pela coordenação do fluxo de entrada e pelo direcionamento para a triagem das unidades, lidando frequentemente com pacientes sintomáticos que não utilizavam máscaras. Juntou-se a isso o fato de receberem EPIs que consideravam incompatíveis com a função e inferiores ao ofertado aos PNS, como máscaras cirúrgicas, e não N95, e capote com gramatura abaixo da indicada pela Agência Nacional de Vigilância Sanitária (Anvisa) para EPI^17^.

Apesar de os protocolos de EPI e segurança reduzirem inseguranças após 2021, o medo da morte persistiu. A preocupação com infectar parentes levou profissionais a adotar medidas de segurança ao chegar em casa, especialmente aqueles com crianças ou idosos. Essas medidas incluíam higienizar as roupas e o corpo antes de entrar e, em casos extremos, enviar crianças e idosos para a casa de parentes.

O medo da morte dos pacientes afetava a saúde mental dos PS, resultando em pânico e em crises de ansiedade. Os ACS que moravam em Manguinhos viam cotidianamente as mortes por Covid-19 de vizinhos e parentes. Isso fez com que o trabalho nas USF "ficasse insustentável":

"[…] no meu caso, eu saí realmente porque eu já estava com o psicológico muuuuito, assim, tinha certeza que eu ia morrer, certeza plena. Eu falei, ‘eu vou morrer’. Ou era isso ou era ter um surto psicótico" (GFA0209).

"Tem gente morrendo […]. E o pânico, né? E a crise de ansiedade já te abalando, e você sem saber o que fazer […]. Foi muito complicado. E a gente na linha de frente, sem saber se a gente […] seria o próximo, a próxima vítima, né?" (GFB0101).

Este fato era exacerbado pelo comportamento dos próprios usuários, que chegavam temerosos da morte, dentro dos serviços de saúde. De acordo com os relatos, os profissionais tinham que tentar "se manter firme" para ajudar, apesar de ao mesmo tempo estarem com "medo de perder a própria vida", e assegurar que os pacientes ficariam bem:

"A gente chegava e tinha um paciente que falava: ‘por favor, não me entuba porque eu não quero morrer’ e no fim do dia ele morria" (ESB03).

A sensação do medo da morte foi diminuída pelo início da vacinação de PS:

"Então, eu me senti muito aliviada quando eu consegui tomar, mas ao mesmo tempo muito pesarosa pelas duas pessoas que eu perdi, que não tiveram oportunidade de tomar. E foi muito próximo, assim, foi coisa de… uma era [a] uma semana de tomar e outro era tipo [a] duas semanas, por aí" (GFA0205).

Apesar disso, muitos participantes relataram que a demora para a compra e disponibilização da vacina também gerava ansiedade:

"A parte da vacinação demorou pra caramba. Então, assim, a gente, pra proteger os profissionais, pra eles, pra eles estarem ali na parte da frente para trabalhar, demorou. Então, assim, foi muita ansiedade, muito tenso, esperar" (ESB02).

No momento de realização da pesquisa, após três anos do início da pandemia, ainda haviam relatos de PNS que não se recuperaram da pressão psicológica dos anos mais intensos da Covid-19:

"A saúde mental dos trabalhadores, realmente, até hoje a gente ainda vê que algumas pessoas não voltaram a sua normalidade, né? […]. A gente está vendo muitas pessoas ainda com pânico, surgindo depressões pós-pandemia… […] A gente está vendo gente que ainda não se recuperou, que adoeceu mesmo" (ESA01).

### Impactos da Pandemia de Covid-19 no Processo de Trabalho das USF

#### "Enquanto a atenção básica, ela acabava vivendo uma sobrecarga gigantesca": sobrecarga de trabalho

Outro fator de repercussão na saúde mental foi o aumento da carga de trabalho no período pandêmico devido ao aumento da jornada, ao afastamento dos profissionais de maior vulnerabilidade e à frequente dupla carga de trabalho (APS e emergências), causando *burnout* em muitos profissionais.

Isso gerou aumento em afastamento/licença, sobrecarregando aqueles que ficavam. Na percepção dos PS, o afastamento de ACS e PNS por comorbidades e infeção pela Covid-19 causou redução das equipes, aumentando a carga de trabalho dos restantes.

Além disso, ACS relataram, no momento da produção dos dados, estarem há anos sem férias pela mudança de organização social contratada pela SMS-RJ. O trabalho contínuo afetou a saúde mental desse grupo, aumentando a pressão que sentiam para conseguirem manter as escalas de trabalho. Aqueles que estavam afastados também sentiram mudanças na rotina para dormir e falta de tempo para o descanso, pois, mesmo em casa, havia a preocupação com os usuários que estavam internados e o lamento por aqueles que haviam morrido. Esses pensamentos fizeram com que os ACS e PNS não conseguissem se desconectar do trabalho.

A incerteza das normas técnicas, que exigiam estudo fora das horas regulares de trabalho para adaptação constante dos PS, aumentou a carga de trabalho. Eram comuns alterações súbitas sobre os critérios para a realização de testes, a forma de recepção dos usuários e o uso de EPIs. Para os ACS, a exaustão física, resultante das longas horas ao ar livre, sob o sol e em pé, para triagem de pacientes nas USF, incluiu relatos de desmaios.

#### "A gente começou a ver uma outra realidade, que a atenção primária não via": sensação de incapacidade

A pandemia fez com que usuários em estado grave buscassem a USF, levando os PS a encararem uma realidade com a qual não estavam preparados. O trabalho extrapolou as funções que os PNS exerciam nos serviços, considerado desgastante e que exigiu conhecimentos para além daqueles relacionados à APS. Era recorrente o atendimento de casos graves, a tomada de decisão para evitar a morte, o acompanhamento para hospitais e a falta de leitos no dia a dia do trabalho. Meses de trabalho nessas condições afetaram negativamente a saúde mental dos PNS:

"Da parte psicológica mesmo, de muito estresse no trabalho. Teve momentos, assim, que a gente tinha que tomar decisões […] que não deveriam ser de atenção primária. […] Já não tava mais sustentando ficar nessa situação e de realmente querer desistir" (ESB04).

A falta de leitos dificultava as internações e fez com que muitos pacientes mais graves fossem tratados na própria USF. De acordo com os relatos, esse tipo de situação foi comum durante 2020 e 2021, sendo agravada nos momentos de pico de internações. Os PS relataram a sensação de estarem perdendo o controle da situação, aumentando sua ansiedade e o estresse no trabalho.

Outro ponto importante foi a falta e/ou dificuldade de ambulâncias para a realização da transferência dos usuários que precisavam de tratamento intensivo, sendo que uma PNS utilizou o próprio carro para conseguir levar uma usuária ao hospital. Em uma das USF houve casos de a equipe de saúde passar a noite com usuários pela falta de leitos de Covid-19 no município. Isso também esgotou os PNS, que fizeram o possível para evitar as mortes de usuários, principalmente na primeira onda.

Além disso, os profissionais foram confrontados com a morte de pacientes. Para os ACS, a morte de usuários na APS foi mais chocante, pois não trabalhavam em hospitais e, por isso, não viam pessoas morrendo de forma recorrente e nem entubadas, gerando implicações na saúde mental desse grupo. Ademais, as pessoas que morriam eram frequentemente amigos ou parentes, outro fator que teve impacto negativo.

A percepção dos PNS foi de que sua função mudou com a pandemia de Covid-19, principalmente pela necessidade de atendimento rápido de usuários que precisavam de primeiros socorros para não morrerem. Aqueles que trabalhavam em hospitais de Covid-19 atendiam mais esses casos, pois tinham maior experiência, e foram comparados a "emergencistas" ou "intensivistas" (fora do escopo da APS). Dessa forma, tais profissionais também repassavam o conhecimento adquirido em hospitais para aqueles que não tinham tais habilidades.

Para os ACS, a pandemia significou uma mudança em seu processo de trabalho, com redução das visitas domiciliares, restritas apenas a grupos de risco e devendo ser breves e restritas ao ambiente peridomiciliar (frente, lados e fundos da casa). Os ACS foram escalados para atividades burocráticas na USF, sob o argumento de que circular no território os tornavam potenciais propagadores do vírus. Isso foi considerado como desvio de função pelos profissionais, que apresentavam certa nostalgia quanto ao tipo de trabalho anterior e ressentiam a "burocratização" de suas funções.

#### "A gente também não teve apoio. A gente não tinha quem procurar": falta de ações de saúde mental

Os relatos conjuntos de PNS e ACS indicam que a SMS-RJ não tratou a saúde mental dos PS como prioridade durante a pandemia de Covid-19. De acordo com os PNS, houve relatos de tentativas por parte da SMS-RJ de oferecer algum suporte, via telemedicina e acompanhamentos, que, em muitos casos, não eram realizados por psicólogos. O apoio com esses profissionais começou a ser ofertado, segundo os PNS, de forma tardia e já no final da pandemia:

"Eu percebi que esse acompanhamento acabou sendo um pouco tardio. Quando se olhou para o profissional, já era mais para o final da pandemia, quando as coisas já estavam diferentes" (ESB01).

As USF criaram espaços e atividades informais para apoiar a saúde mental dos PS, como conversas com colegas. No entanto, essas iniciativas não foram institucionalizadas. As poucas atividades de cuidado em saúde mental disponíveis não foram amplamente acessadas pelos PNS, devido às escalas de trabalho que dificultavam a busca por atenção especializada.

Os ACS relatam que se sentiram excluídos dos cuidados de saúde mental, pois só recebiam as informações sobre atividades posteriormente. Para eles, isso exacerbava a questão mais profunda de não serem considerados como PS plenos.

"Disseram que tinham telefones, tinham psicólogos *online*, diversas ações que estavam disponíveis e que nós só fomos chamados para apresentação. Mas não fomos citados e nem [foram] utilizados para nós" (GFA0113).

## DISCUSSÃO

Os resultados do nosso estudo apontaram que a sobrecarga de trabalho, o medo de infecção, a falta de recursos e a perda de controle sobre o ambiente de trabalho contribuíram para o sofrimento mental entre PS durante a pandemia de Covid-19. Evidencia-se, ainda, a necessidade de disponibilizar apoio psicológico, treinamento em EPI e promoção de mudanças na organização do trabalho desde o início de uma crise sanitária.

Verificamos, ainda, que os efeitos da pandemia de Covid-19 sobre a saúde mental de PS têm desdobramentos mesmo após seu fim, assim como encontrado em estudos internacionais^18-20^.O sofrimento psicológico dos PS pode impactar, além do bem-estar, o desempenho profissional, a qualidade do atendimento e a segurança dos usuários dos serviços^18^. A repercussão dos efeitos de saúde mental é um fator importante que deve ser considerado no planejamento de políticas de saúde dos PS no Rio de Janeiro.

Estudos no Brasil^7,8,21^ e em outros países^11,22,23^ também apontaram que o medo da morte foi um fator crucial, agravado pela percepção de limitação de EPI, pela falta de testes de Covid-19 para os PS^7,8,24^, e pela demora na vacinação. Assim, a escassez ou má qualidade de EPI, principalmente entre ACS, potencializou a ansiedade vinculada à possibilidade de infecção. A proximidade com o sofrimento e a morte dos pacientes levou ao sofrimento psíquico e ao adoecimento mental dos PS^8,24,25^, potencializando um sentimento de responsabilidade profissional e cívica entre os PS^18,26^. Corroborando os resultados desta pesquisa, o estudo de revisão encontrou que o medo de infectar um ente querido foi uma das principais fontes de angústia durante a pandemia de Covid-19, sendo destacado por 15 artigos^6^.

A sobrecarga de trabalho também impactou negativamente a saúde mental dos PS^27^, principalmente quando assumiam diversas funções relacionadas ao controle da pandemia^22,27,28^, indicando que o problema não é apenas o contato com o vírus, mas o trabalho excessivo. Aliada à sobrecarga estava a sensação de estar perdendo o controle da situação, principalmente em relação à gravidade dos pacientes, o que também foi encontrado em estudo realizado no Irã^2^. O grande número de pacientes, a elevada taxa de hospitalizações, a alta mortalidade e o fato de não haver nada que pudessem fazer para salvar esses pacientes aumentaram a sensação de perda de controle^7^.

No que diz respeito ao processo de trabalho, as fontes de sofrimento diferiram entre PNS e ACS. Enquanto os PNS sofreram com a adaptação à gravidade dos casos, a necessidade de adquirir rapidamente novos conhecimentos e a sensação de perda de controle, os ACS sentiram maior burocratização de seu trabalho, com mudanças em suas funções. Um achado importante foi a sensação de exclusão dos ACS da categoria de PS. Existem estudos que apontam que os ACS se sentem o elo mais fraco da equipe multidisciplinar e pouco reconhecidos profissionalmente pelas gerências e SMS^29^, porém o assunto é pouco explorado. Além disso, os ACS relataram que problemas pré-existentes, como a precarização das relações trabalhistas, se intensificaram durante a pandemia^29^.

Os resultados do nosso estudo sobre os PS da APS evidenciam desafios semelhantes aos enfrentados por PS de urgência e emergência na pandemia. Assim como na APS, profissionais de emergência relataram sobrecarga de trabalho, medo de infecção e insuficiência de recursos para tratamento dos pacientes, fatores que intensificaram o sofrimento mental^30^. Entretanto, existem diferenças notáveis nas experiências relatadas por PS em diferentes contextos. Enquanto os trabalhadores da APS enfrentaram desafios significativos devido à escassez de EPIs e à desvalorização profissional, os profissionais em unidades de emergência frequentemente lidaram com sobrecarga de estresse devido ao atendimento contínuo a pacientes críticos^31,32^.

Como limitações, destacam-se: o fato de a coleta de dados ter sido realizada apenas no Complexo de Manguinhos, no Rio de Janeiro; a ausência de GF com PNS, pois isso pode ter restringido a diversidade de perspectivas coletadas, especialmente em temas relacionados a gestão e práticas clínicas; o predomínio de ACS na amostra, grupo que apresenta diferenças significativas dos PNS quanto à raça e escolaridade, provendo maior peso às experiências e aos desafios vivenciados por esse grupo; a coleta de dados após os períodos mais críticos da pandemia pode ter influenciado as percepções relatadas, refletindo um momento de adaptação e recuperação, ao invés das situações mais agudas enfrentadas durante a crise.

Nossos resultados evidenciaram a necessidade de se priorizar a saúde mental dos PS desde o início de uma crise sanitária. Preparar-se para futuras emergências de saúde implica abordar essas questões de forma proativa, minimizando fatores de estresse com adequada distribuição de carga de trabalho, fornecimento de EPI e acesso a serviços gratuitos de saúde mental. A experiência com a Covid-19 mostra que a preparação para emergências sanitárias deve ser contínua, com planos de contingência baseados em evidências, coordenação, comunicação, apoio aos trabalhadores e investimento na produção de insumos.

## Disponibilidade de Dados:

Os dados estão disponíveis mediante solicitação ao autor correspondente.
